# Research and Performance Evaluation of Environmentally Friendly Shale Inhibitor TIL-NH_2_ for Shale Gas Horizontal Wells

**DOI:** 10.3390/molecules29245950

**Published:** 2024-12-17

**Authors:** Yuexin Tian, Xiangjun Liu, Yintao Liu, Haifeng Dong, Guodong Zhang, Biao Su, Xiaofeng Liu, Yifan Hu, Jinjun Huang, Zeze Lu

**Affiliations:** 1 Petroleum Engineering Technology Institute of Southwest Petroleum Branch, SINOPEC, Deyang 618000, China; liuyintao.xnyq@sinopec.com (Y.L.); donghaifeng.xnyq@sinopec.com (H.D.); zhangguodong.xnyq@sinopec.com (G.Z.); subiao.xnyq@sinopec.com (B.S.); liuxiaofeng.xnyq@sinopec.com (X.L.); huyf2022.xnyq@sinopec.com (Y.H.); 2 State Key Laboratory of Oil and Gas Reservoir Geology and Exploitation, Southwest Petroleum University, Chengdu 610500, China; 13880093092@163.com (X.L.); huangjjswpu@163.com (J.H.); 3 Changqing Down Hole Technology Company, CCDC, Xi’an 710000, China; 18200236700@163.com

**Keywords:** shale, wellbore stability, inhibitor, inhibitory performance, environmental friendliness

## Abstract

Wellbore instability caused by hydration during the development of shale gas reservoirs poses significant challenges to drilling engineering. In this study, a novel and environmentally friendly shale inhibitor, TIL-NH_2_, was synthesized via free radical polymerization using 1-vinylimidazole and N-(2-bromoethyl)-1,3-propanediamine dihydrobromide as the main raw materials. The molecular structure of TIL-NH_2_ was characterized by infrared spectroscopy and nuclear magnetic resonance. Incorporating imidazole cations and amino bifunctional groups, TIL-NH_2_ exhibits excellent inhibitory performance and environmental friendliness. Its performance was systematically evaluated through linear swelling tests, shale cuttings rolling recovery tests, permeability recovery experiments, and dynamic adsorption analyses. The results indicate the following: (1) At a concentration of 1.2 wt%, TIL-NH_2_ reduced the linear swelling height of shale by 65.69%, significantly outperforming traditional inhibitors like KCl and NW-1. (2) Under conditions of 140 °C, the cuttings rolling recovery rate of TIL-NH_2_ reached 88.12%, demonstrating excellent high-temperature resistance. (3) Permeability recovery experiments showed that at a concentration of 2.0 wt%, TIL-NH_2_ achieved a permeability recovery rate of 90.58%, effectively mitigating formation damage. (4) Dynamic adsorption experiments indicated that at a concentration of 2.5 wt%, the adsorption capacity tended toward saturation, reaching 26.00 mg/g, demonstrating stable adsorption capability. Additionally, environmental friendliness evaluations revealed that TIL-NH_2_ has a degradation rate exceeding 90% within 28 days, and its acute toxicity is significantly lower than that of traditional inhibitors like KCl (the LC_50_ of TIL-NH_2_ is 1080.3 mg/L, whereas KCl is only 385.4 mg/L). This research provides a high-efficiency and environmentally friendly new inhibitor for green drilling fluid systems in horizontal shale gas wells, offering important references for technological advancements in unconventional energy development.

## 1. Introduction

As an important unconventional natural gas resource, shale gas has abundant reserves and enormous development potential, making it a key direction in the adjustment of the global energy structure [1,2,3]. Shale gas reservoirs are characterized by low porosity and low permeability, and their development mainly relies on horizontal drilling and fracturing technologies. However, shale formations are prone to wellbore instability during drilling, primarily because water-based drilling fluids infiltrate the shale formations, leading to shale hydration swelling and the peeling and dispersion of clay minerals. This phenomenon not only affects wellbore stability but also leads to complex wellbore issues, increasing drilling difficulty and costs [4,5,6,7]. To address this problem, developing shale inhibitors with high-efficiency inhibitory performance has become a research hotspot.

For a long time, the influence and control of water-rock interactions on the mechanical properties of rock masses have been important research directions in the field of petroleum engineering, both domestically and internationally [8,9,10]. Previous studies have mostly focused on hydrating and swelling mudstones rich in montmorillonite, while systematic analyses of the hydration behavior of shales rich in illite—a weakly swelling clay mineral—and its impact on shale structural stability are still insufficient. This limitation directly affects the safety and technical efficiency of oil and gas development in shale formations. Therefore, exploring new hydration inhibitors to effectively control shale hydration swelling has become a key technological means to promote continuous progress in oil and gas development [11,12,13]. 

Currently, significant progress has been made in the research of inhibitors such as inorganic salts [14,15,16], organic salts [17,18], surfactants [19,20], natural product modifications [21,22,23], and polymers [24,25,26]. Among them, polymer inhibitors have received widespread attention in the field of shale hydration inhibition due to their excellent adsorption and coating abilities and good thermal stability. In recent years, research on polymer ionic liquid inhibitors has become a hotspot. Water-based drilling fluid systems using polymer ionic liquids as core treatment agents have performance close to that of oil-based drilling fluids [27,28,29] and have been widely applied in oilfields both domestically and abroad. Additionally, cross-linked polymers and hyperbranched polymers, due to their special spatial structures, have significant advantages in inhibiting shale hydration swelling and dispersion [30]. However, existing inhibitors still have shortcomings in high-temperature and high-salinity resistance, environmental friendliness, and multifunctionality, making it difficult to fully meet the demands of complex environments in deep and ultra-deep wells.

In the broader context of developing environmentally friendly shale inhibitors, recent studies have begun to explore bio-based and biodegradable polymers (e.g., chitosan, cellulose derivatives) that reduce environmental impacts, as well as nanotechnology-enhanced inhibitors incorporating materials such as graphene and nano-silica to improve adsorption and stability [31,32,33,34]. Furthermore, novel ionic liquid-based inhibitors and hybrid polymeric materials have shown promising lab-scale results, offering improved performance under harsh downhole conditions with reduced ecological footprints [35,36,37,38]. These emerging directions underscore the industry’s pursuit of “green” shale inhibitors that not only maintain or improve mechanical stability but also meet increasingly stringent environmental standards.

In response to these issues, the design and development of inhibitors need to balance inhibitory performance with actual engineering requirements. To enhance inhibitory effectiveness, endowing inhibitors with multifunctionality has become an important trend in polymer inhibitor research. Recently, we reported the synthesis and characterization of a novel polyionic liquid shale inhibitor, TIL-NH_2_ [39]. That study focused on the synthesis route, structural characterization, and the underlying inhibition mechanism of TIL-NH_2_ through various techniques such as infrared spectroscopy, NMR spectroscopy, and X-ray diffraction. Building upon this foundation, the present study aims to provide a comprehensive performance evaluation of TIL-NH_2_ under simulated downhole conditions. This includes evaluating its inhibition efficiency in linear swelling tests, cuttings rolling recovery tests, and its impact on shale permeability, particularly under high-temperature conditions, which are crucial for deep shale gas exploration. Unlike conventional inhibitors that typically rely on a single functional group and often struggle to maintain performance in harsh downhole conditions, TIL-NH_2_’s bifunctional architecture enables enhanced adsorption and coating effects under high-temperature and high-salinity environments. Furthermore, its rapid biodegradability and significantly reduced acute toxicity mark a substantial step forward in environmental responsibility. These attributes set TIL-NH_2_ apart from traditional options such as KCl, providing a more sustainable and robust solution for field applications.

This study not only provides an environmentally friendly inhibitor with excellent performance for shale gas horizontal well drilling fluid systems but also offers scientific basis and technical support for the development of new shale inhibitors from the perspectives of molecular design, performance optimization, and environmental protection. It provides a solid foundation for the green development and low-cost, efficient exploitation of shale gas.

## 2. Results

### 2.1. Preparation of the Inhibitor TIL-NH_2_

In 70 mL of acetonitrile solvent, 12.3 g of 1-vinylimidazole was added, and the mixture was poured into a three-necked flask equipped with a reflux condenser. The system temperature was raised to 80 °C, after which 25.6 g of N-(2-bromoethyl)-1,3-propanediamine dihydrobromide was added. The reaction mixture was stirred and refluxed at 80 °C for 24 h. Upon completion of the reaction, triethylamine was added to remove HBr from the reaction mixture, forming triethylamine hydrobromide. Vacuum filtration was then performed, and the resulting precipitate was washed multiple times with anhydrous ethanol. Finally, the precipitate was dried in a vacuum oven at 45 °C for 24 h to obtain a light orange solid product—this is the synthesized monomer (YX-NH_2_) with a yield of 84.2%.

**Figure 1:**
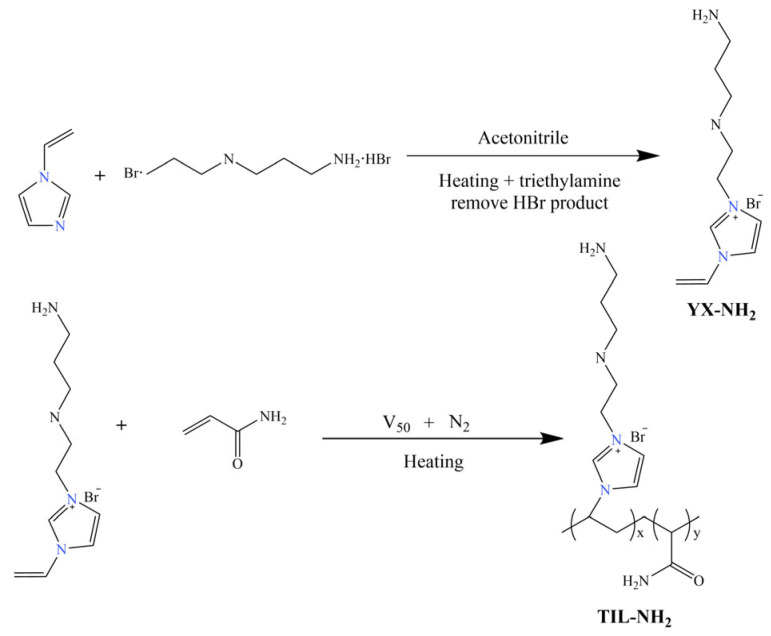
Reaction mechanism equation.

An appropriate amount of YX-NH_2_ monomer and acrylamide was dissolved in deionized water and placed into a three-necked flask equipped with a reflux condenser. The pH was adjusted to 5, and the temperature was raised to the desired level under reflux conditions. A small amount of V-50 initiator was added under a nitrogen atmosphere. The polymerization reaction was carried out under stirring at 200 r/min while maintaining a constant temperature for several hours. After the reaction was completed, the mixture was transferred to a rotary evaporator for vacuum distillation for 2.5 h. An orange-red viscous solid product was obtained, which is the polymer ionic liquid TIL-NH_2_. The reaction mechanism is shown in Figure 1.

### 2.2. Physicochemical Characterization of TIL-NH_2_

#### 2.2.1. Infrared Spectroscopy

**Figure 2:**
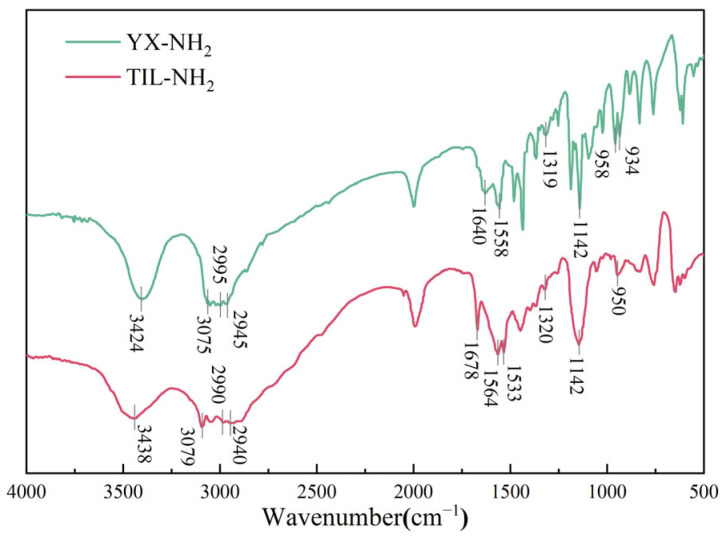
TIL-NH_2_ inhibitor infrared spectra [39].

The IR spectral results of TIL-NH_2_ are shown in Figure 2.

Figure 2 presents the infrared spectra of the YX-NH_2_ monomer and the TIL-NH_2_ polymer. In the spectrum of YX-NH_2_, the broad band at 3424 cm^−1^ corresponds to the fundamental stretching vibration peak of the N-H bond in the primary amine functional group. The peak at 3075 cm^−1^ is attributed to the fundamental stretching vibration of the C-H bond in the imidazole ring. Peaks at 2995 cm^−1^ and 2940 cm^−1^ are assigned to the fundamental stretching vibrations of the C-H bonds in the methyl and methylene groups of the side chain. The peak at 1640 cm^−1^ corresponds to the fundamental stretching vibration of the C=C bond in the vinyl group. The peak at 1558 cm^−1^ is associated with the fundamental stretching vibration of the N=C bond in the cationic imidazole ring. The peak at 1319 cm^−1^ represents the fundamental skeletal vibration of the imidazole ring. The peak at 1142 cm^−1^ corresponds to the fundamental in-plane bending vibration of the C-H bond in the imidazole ring. The peak at 958 cm^−1^ is attributed to the fundamental stretching vibration of the imidazole ring, and the peak at 934 cm^−1^ corresponds to the fundamental in-plane rocking vibration of the C-H bond in the propenyl group.

These spectral characteristics indicate that the YX-NH_2_ monomer possesses a primary amine functional group, a cationic imidazole five-membered ring structure, and a vinyl group. Under the initiation of V-50, the vinyl groups of the YX-NH_2_ monomer are attacked by free radicals, undergoing a free radical polymerization reaction to form the TIL-NH_2_ polymer. In the infrared spectrum of TIL-NH_2_, aside from the spectral features of the primary amine functional group and the cationic imidazole five-membered ring remaining unchanged, a new peak appears at 1678 cm^−1^, which is the fundamental stretching vibration peak of the carbonyl C=O bond in the amide group (-CONH_2_). Meanwhile, the spectral features at 1640 cm^−1^ corresponding to the C=C bond of the vinyl group and at 934 cm^−1^ corresponding to the C-H bond disappear. This indicates that the double bonds of the vinyl groups have opened, resulting in the formation of a long-chain polymer structure.

#### 2.2.2. Nuclear Magnetic Resonance Spectral Analysis

**Figure 3:**
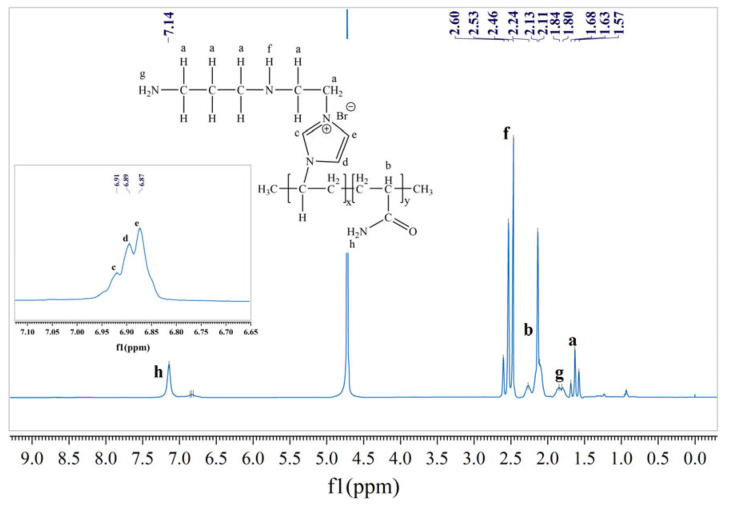
^1^H-NMR spectrum of TIL-NH_2_ [39].

To more accurately characterize the structure of TIL-NH_2_, proton nuclear magnetic resonance (^1^H-NMR) spectroscopy was performed. The ^1^H-NMR spectra were recorded on a Bruker AVANCE III NMR spectrometer operating at 500 MHz. A standard one-dimensional pulse sequence with a relaxation delay (d1) of 2 s and an acquisition time (AQ) of approximately 3 s was employed. The sample was prepared by dissolving approximately 12 mg of TIL-NH_2_ in 0.5 mL of deuterated dimethyl sulfoxide (DMSO-d6, 99.9% purity), and tetramethylsilane (TMS) was used as the internal standard. The solution was gently filtered through a 0.45 μm PTFE filter to remove any particulate matter prior to analysis. All measurements were performed at ambient temperature (25 ± 1 °C), and data processing was carried out using Bruker TopSpin software (version 4.4.0). The obtained ^1^H-NMR spectrum is shown in Figure 3, where the letters labeling the chemical shift peaks correspond to the hydrogen atoms at the designated positions in the chemical structure.

As illustrated in Figure 3, the ^1^H-NMR spectrum of TIL-NH_2_ clearly displays distinct chemical shifts corresponding to labeled protons (a–h): 1.57–1.68 ppm (a) is assigned to the hydrogen atoms of the -CH_2_- groups on the polymer backbone; 2.11–2.24 ppm (b) corresponds to the hydrogen atoms of the -CH- groups in the acrylamide structural units on the polymer backbone; 6.67–6.91 ppm (c, d, e) is attributed to the hydrogen atoms on the imidazole rings of the polymer side chains; 2.48–2.60 ppm (f) originates from the hydrogen atoms in the secondary amine (-NH-) groups; 1.63–1.84 ppm (g) is assigned to the hydrogen atoms in the primary amine (-NH_2_) groups; and 6.80–7.14 ppm (h) corresponds to the hydrogen atoms in the amide (-CONH_2_) groups. These labeled positions (a–h) in Figure 3 represent distinct proton environments in the polymer structure, providing clear insight into the chemical composition of TIL-NH_2_.

Based on the analysis of these chemical shifts, it is evident that the synthesized product is the target compound TIL-NH_2_. Compared to the broad absorption bands of primary and secondary amines at 3200–3400 cm^−1^ in the infrared (IR) spectrum, the ^1^H-NMR spectrum more precisely distinguishes between the primary and secondary amine functional groups present in TIL-NH_2_.

### 2.3. Performance Evaluation of TIL-NH_2_

#### 2.3.1. Linear Expansion Experiments

**Figure 4:**
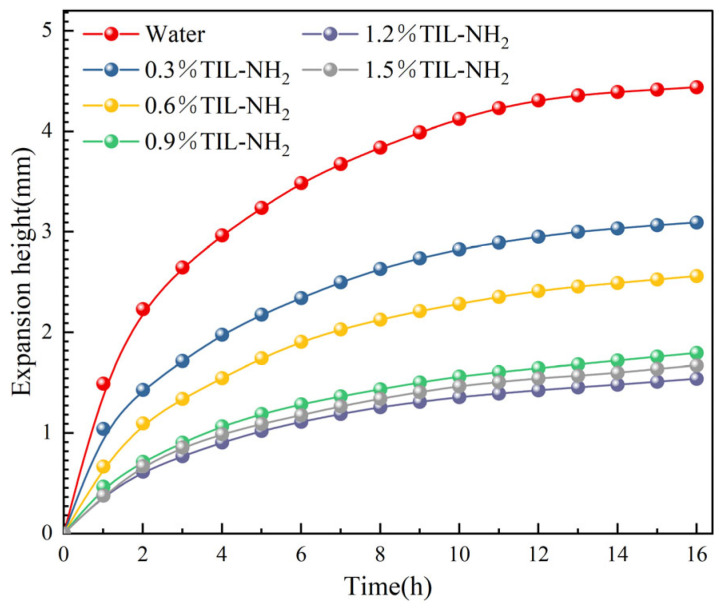
Relationship between shale swelling height and immersion time in TIL-NH_2_ solutions with different concentrations.

To quantitatively evaluate the inhibitory effect of TIL-NH_2_ on shale hydration swelling, linear swelling rate tests were conducted on shale powder using TIL-NH_2_ solutions of varying concentrations. The concentrations of TIL-NH_2_ solutions tested were 0.3%, 0.6%, 0.9%, 1.2%, and 1.5%. Simultaneously, linear swelling rate tests were performed using 5% KCl, 2% NW-1, and 2% polyetheramine solutions for comparison and analysis. The effect of different concentrations of TIL-NH_2_ solutions on the linear swelling rate is illustrated in Figure 4. (Experimental data points are represented by circles. The solid lines are simple interpolation lines used to illustrate general trends and do not represent any mathematical models or fitting functions.).

Figure 4 shows the inhibitory effects of aqueous TIL-NH_2_ solutions at different concentrations on the linear swelling of shale powder. Except for the 0.3% TIL-NH_2_ solution, other concentrations significantly reduced the hydration swelling height of the shale powder. When the concentrations of TIL-NH_2_ were 0.6%, 0.9%, 1.2%, and 1.5%, the swelling heights after 16 h were 2.55 mm, 1.79 mm, 1.52 mm, and 1.66 mm, respectively. Compared to the 4.43 mm swelling height in distilled water, these represent reductions of 42.44%, 59.59%, 65.69%, and 62.53%, respectively, with the 1.2% TIL-NH_2_ solution exhibiting the best inhibitory effect. This indicates that TIL-NH_2_ has an efficient ability to inhibit shale hydration swelling, which may be related to its bifunctional structure. TIL-NH_2_ contains not only primary amine groups but also imidazole cationic groups. The latter is a functional group with catalytic activity and hydrogen-bond-forming ability, which can interact with functional groups on the shale surface, thereby inhibiting its hydration swelling.

**Figure 5:**
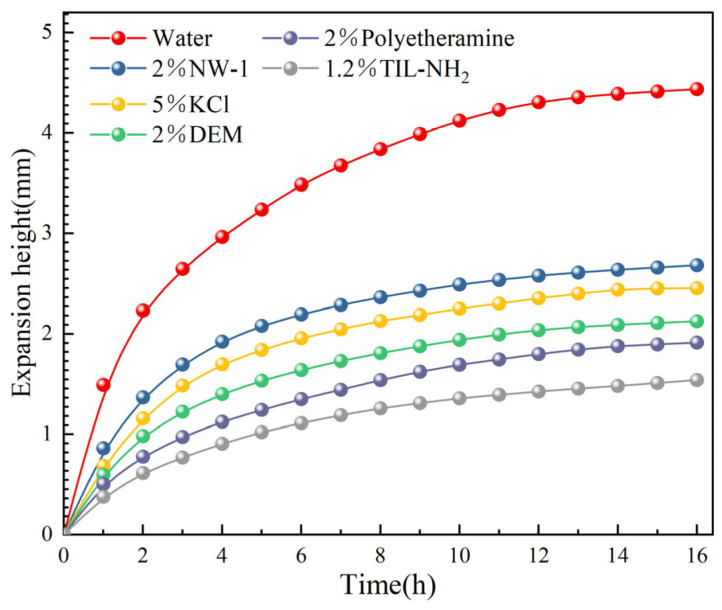
Relationship between shale swelling height and immersion time under different concentrations of inhibitor solution.

Figure 5 presents the effects of different inhibitor solutions on the linear swelling of shale powder. (Experimental data points are represented by circles. The solid lines are simple interpolation lines used to illustrate general trends and do not represent any mathematical models or fitting functions). Compared with the distilled water group, all inhibitor solutions effectively reduced the hydration swelling height of the shale powder. After 16 h, the swelling heights for the KCl and NW-1 groups were 2.44 mm and 2.68 mm, respectively, compared to 4.43 mm for the distilled water group, indicating that both have similar inhibitory effects. In the early swelling stage, the swelling height of the 2% polyetheramine group was slightly lower than that of the 2% polyamine DEM group, but after 16 h, the difference between the two groups was not significant. In the linear swelling experiments, the best inhibitor was the 1.2% TIL-NH_2_ group, which maintained swelling rates lower than those of other inhibitor groups within 16 h. The swelling height after 16 h was only 1.52 mm, demonstrating a significant inhibitory effect.

#### 2.3.2. Rock Chip Rolling Recovery Experiment

**Figure 6:**
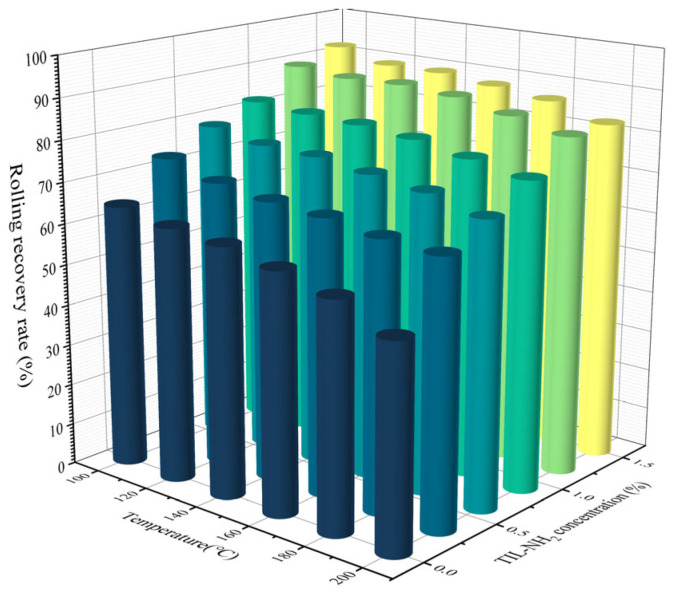
Variation in heat rolling recovery with TIL-NH_2_ addition at different temperatures.

To effectively control the hydration effect of drilling fluids on shale cuttings and considering the influence of geological conditions on shale hydration, the thermal rolling recovery rate was used under simulated high-temperature conditions in the laboratory to evaluate the inhibitory performance of TIL-NH_2_. The experimental results are shown in Figure 6.

Figure 6 reveals the impact of TIL-NH_2_ concentration on the thermal rolling recovery rate of shale under different temperature conditions. Compared with the distilled water group, the addition of TIL-NH_2_ significantly inhibited the hydration and dispersion of shale cuttings, thereby increasing the thermal rolling recovery rate. Even at lower concentrations, TIL-NH_2_ achieved relatively high recovery rates; when the concentration exceeded 0.9%, the recovery rates at all temperatures were above 75%. At a concentration of 1.2%, the thermal rolling recovery rates exceeded 82% within the temperature range of 120–180 °C.

This enhanced performance may be attributed to the presence of two inhibitory functional groups in TIL-NH_2_. The dual functional groups enhance the adsorption and encapsulation capabilities of TIL-NH_2_, effectively preventing the hydration and dispersion of shale. The side chains of TIL-NH_2_ contain imidazole cationic structures, which remain structurally stable even at temperatures up to 300 °C. This stability allows TIL-NH_2_ to maintain its inhibitory effect, resulting in a gradual decrease in the thermal rolling recovery rate rather than a sharp decline.

Even under the highest temperature condition of 200 °C, the 0.9% concentration of TIL-NH_2_ still exhibited commendable performance, achieving a thermal rolling recovery rate of 75.26%. The excellent high-temperature resistance of TIL-NH_2_ imparts significant application potential, especially in deep and ultra-deep well drilling operations where high-temperature conditions are prevalent.

**Figure 7:**
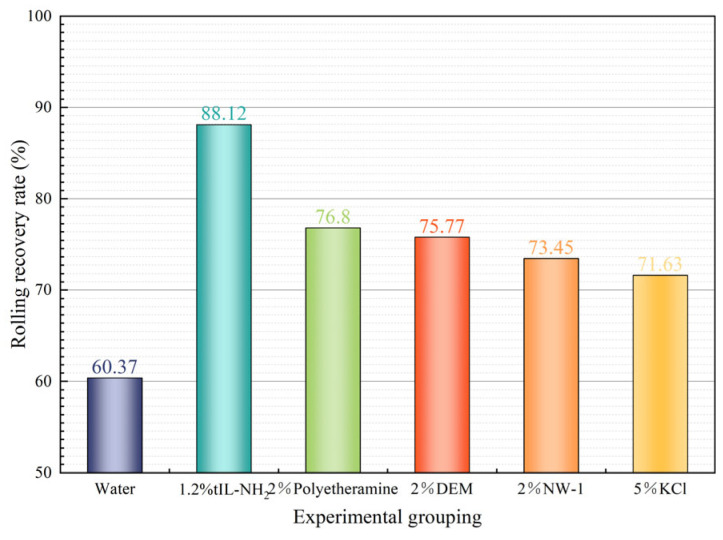
Heat roll recovery for each inhibitor at 140 °C.

Figure 7 compares the thermal rolling recovery rates of shale at 140 °C using different inhibitors, with the concentration of TIL-NH_2_ set at 1.2%. Compared to the distilled water group, the inhibitory effects of various inhibitors on the hydration and dispersion of shale cuttings show noticeable differences. Unlike small-molecule salts such as KCl and NW-1, polyamine inhibitors like DEM, polyetheramine, and TIL-NH_2_ effectively prevent cuttings dispersion due to the encapsulation and adsorption effects of their molecular chains.

Specifically, a 2% polyetheramine solution increased the thermal rolling recovery rate of shale from 60.37% (in distilled water) to 76.80%. The polyamine inhibitor DEM, owing to its hyperbranched structure and high content of polyamine functional groups, can effectively adsorb and encapsulate the cuttings. This action inhibits their hydration and dispersion, resulting in a higher thermal rolling recovery rate. Compared to polyamine inhibitors like DEM and polyetheramine, TIL-NH_2_ possesses a higher molecular weight and dual inhibitory functional groups. These features enhance its adsorption and encapsulation capabilities, enabling it to achieve a recovery rate of 88.12% at a concentration of 1.2%.

#### 2.3.3. Inhibitory Properties of Inhibitors on Ilmenite Hydration

Illite is a layered silicate mineral whose interlayer spaces are occupied by poorly hydrated K^+^ ions, making it less susceptible to hydration swelling. However, when illite comes into contact with water, it still generates a certain degree of hydration swelling stress, leading to the formation and propagation of secondary cracks in shale. Therefore, a key indicator for evaluating the performance of hydration inhibitors is their ability to suppress the hydration swelling stress of illite. In this study, TIL-NH_2_ was used as a hydration inhibitor to measure its effect on the hydration swelling stress of illite.

**Figure 8:**
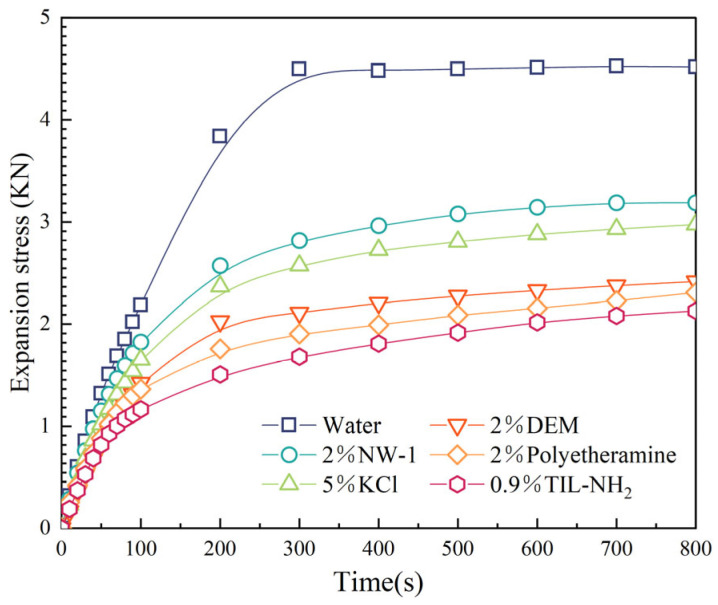
Expansion stress of illite in response to different solution treatments.

Figure 8 presents the hydration swelling stress curves of illite in deionized water and in TIL-NH_2_ solutions of different concentrations. (Experimental data points are represented by descriptions of colors and shapes. The solid lines are simple interpolation lines used to illustrate overall trends and are not based on any mathematical models or fitting functions.) As shown in the figure, TIL-NH_2_ solutions significantly reduce both the hydration swelling stress and the swelling rate of illite. Specifically, a 0.9% TIL-NH_2_ solution reduces the hydration swelling stress of illite by 52%. In contrast, other inhibitors such as NW-1, KCl, DEM, and polyetheramine exhibit weaker inhibitory effects, reducing the hydration swelling stress of illite by 25.4%, 28.9%, 42.7%, and 46.5%, respectively. This indicates that TIL-NH_2_, as a linear polymer containing both imidazole cations and amino functional groups in its molecular structure, can efficiently inhibit the hydration swelling of illite, thereby enhancing shale stability.

**Figure 9:**
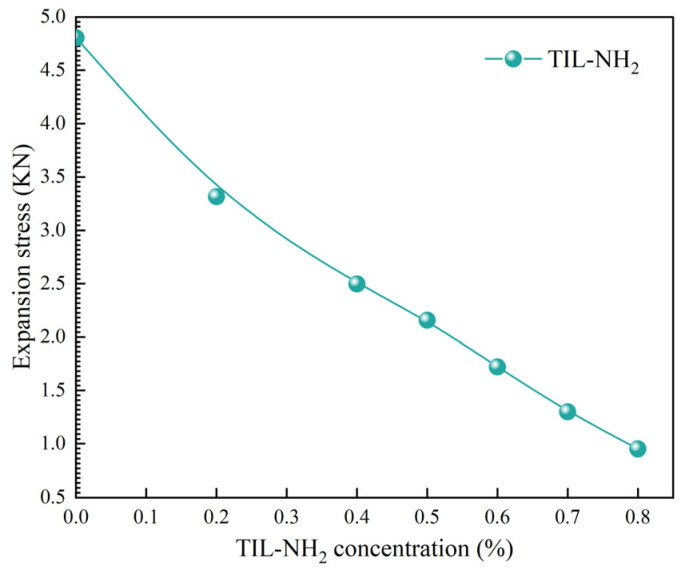
Influence of TIL-NH_2_ concentration on the swelling stress of illite.

The hydration swelling stress of illite refers to the internal stress generated when water molecules occupy the interlayer spaces of illite upon exposure to water, leading to increased interlayer spacing and volume expansion. The polymer inhibitor TIL-NH_2_ can adsorb onto the surface or interlayers of illite, forming a protective film that prevents water molecules from entering, thus inhibiting the hydration swelling of illite. Figure 9 illustrates the effect of TIL-NH_2_ concentration on the hydration swelling stress of illite. (Experimental data points are represented by circles. The solid lines are simple interpolation lines used to illustrate general trends and do not represent any mathematical models or fitting functions). It is evident that there is a negative correlation between the concentration of TIL-NH_2_ and the hydration swelling stress of illite; that is, the higher the concentration, the lower the hydration swelling stress. This is because higher concentrations of TIL-NH_2_ more effectively prevent the ingress of water molecules, reducing the degree of illite hydration.

However, when the concentration of TIL-NH_2_ is too high, its solubility and adsorption capacity decrease, leading to the formation of aggregates in solution. This reduces the contact area with illite and diminishes the inhibitory effect. Therefore, it is necessary to select an appropriate polymer concentration to achieve the optimal inhibitory effect. In this study, a TIL-NH_2_ concentration of 0.6% was chosen to investigate its combined effect with different concentrations of KCl. KCl is a commonly used inhibitor that reduces the hydration capacity of illite by increasing the ionic strength of the solution.

**Figure 10:**
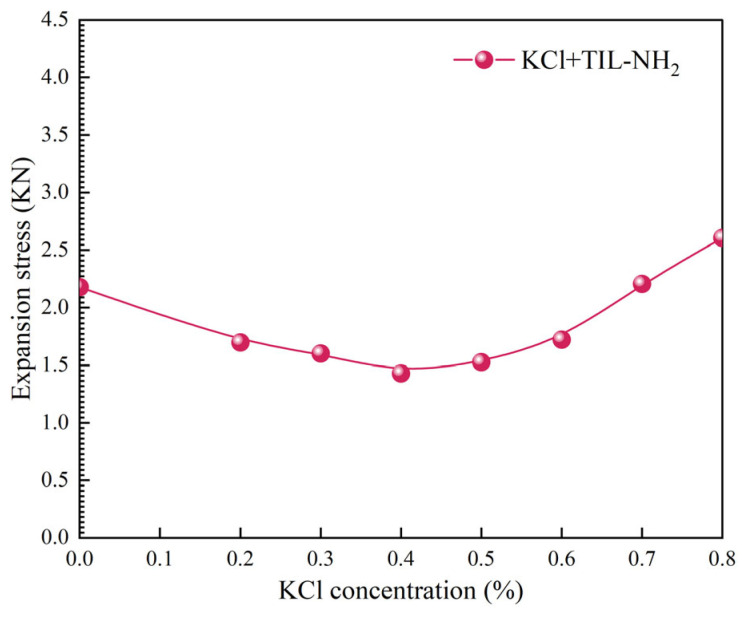
Effect of combined KCl/TIL-NH_2_ solutions on illite swelling stress.

Figure 10 shows the hydration swelling stress curves of illite in the blended solutions. (Experimental data points are represented by circles. The solid lines are simple interpolation lines used to illustrate general trends and do not represent any mathematical models or fitting functions). It can be observed that when the concentration of KCl is low, KCl synergistically interacts with TIL-NH_2_, enhancing the inhibitory effect and reducing the hydration swelling stress of illite by 63%. However, when the concentration of KCl is too high, KCl competes with TIL-NH_2_ for adsorption sites, weakening the inhibitory effect and increasing the hydration swelling stress. Therefore, selecting an appropriate concentration of KCl is essential to achieve the optimal synergistic effect. In this section, a KCl concentration of 0.4% was selected for blending, which achieved the best inhibitory performance.

#### 2.3.4. Effect of Inhibitors on Shale Compressive Strength

In accordance with the GB/T 29172-2012 standard [40] “Core Analysis Methods”, over 50 cylindrical core samples (φ25 mm × 50 mm) were extracted from downhole shale specimens of the Longmaxi Formation in the Weiyuan area. Triaxial compressive strength tests were conducted to investigate the impact of different soaking fluids on the compressive strength of shale. The triaxial compressive strength refers to the maximum shear strength a core sample attains under different normal stresses applied in three directions, leading to failure.

**Figure 11:**
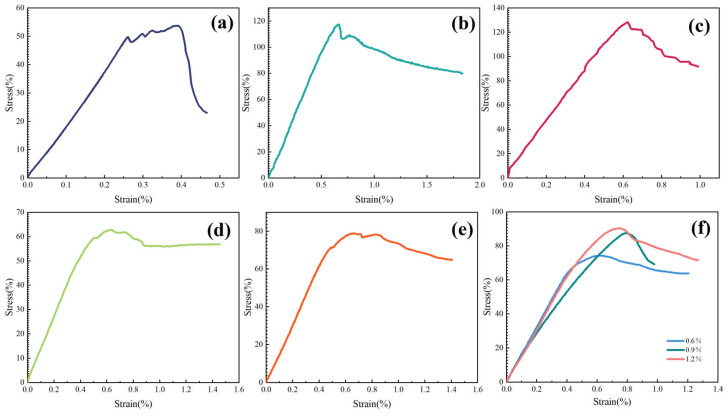
Triaxial stress diagrams of downhole shale of the Longmaxi Formation soaked by different treatments ((**a**) water, (**b**) diesel fuel, (**c**) white oil, (**d**) 2% DEM, (**e**) 2% polyetheramine, (**f**) TIL-NH_2_ solution).

In this section, the triaxial compressive strengths of shale cores soaked for 24 h in water, diesel oil, and white oil were measured. The experimental results are presented in Figure 11.

Figure 11 shows that after soaking in water, the triaxial compressive strength of the Longmaxi Formation shale cores rapidly decreased to 53.7 MPa, a reduction of 92.3 MPa compared to the original core strength. In contrast, cores soaked in diesel oil and white oil exhibited triaxial compressive strengths of 117.6 MPa and 128.3 MPa, respectively—an increase of 63.9 MPa and 74.6 MPa compared to the water-soaked cores.

Shale cores soaked in 2% DEM and 2% polyetheramine solutions had compressive strengths of 62.6 MPa and 79.0 MPa, representing reductions of 83.4 MPa and 67.0 MPa compared to the original cores, but increases of 8.9 MPa and 25.3 MPa relative to the water-soaked samples. Meanwhile, cores soaked in TIL-NH_2_ solutions at concentrations of 0.6%, 0.9%, and 1.2% displayed triaxial compressive strengths of 74.4 MPa, 87.2 MPa, and 90.5 MPa, respectively. These values correspond to reductions of 71.6 MPa, 58.8 MPa, and 55.5 MPa compared to the original cores, but increases of 20.7 MPa, 33.5 MPa, and 36.8 MPa over the water-soaked samples.

These results indicate that soaking in aqueous solutions leads to the lowest maximum differential stress in shale, demonstrating that water reduces the triaxial compressive strength of shale. However, the addition of the inhibitor TIL-NH_2_ partially restores the shale’s compressive strength. This restoration is attributed to the inhibitor’s suppression of shale hydration. The imidazole cations in TIL-NH_2_ molecules reduce the interlayer spacing of clay minerals through electrostatic adsorption and hydrogen bonding interactions. By enhancing the hydrophobicity of the clay surfaces, TIL-NH_2_ effectively slows the ingress of water molecules, thereby curbing the hydration swelling of the clay.

This study demonstrates that the TIL-NH_2_ inhibitor can mitigate the internal damage progression within shale and enhance its compressive strength capacity.

#### 2.3.5. Effect of Inhibitors on Acoustic Time Differences in Shale Cores

The basic data of the rock samples in this section are presented in Table 1, and the test results of the acoustic transit time of the cores are shown in Table 2. The mass fractions of the 1.2 mol/L NaCl, KCl, and CaCl_2_ solutions are 8.05%, 10.23%, and 13.25%, respectively.

Based on the data in Table 1, it is evident that soaking the rocks in solutions caused an increase in mass, indicating that water successfully penetrated the interior of the rocks. However, after drying, the mass of the rock samples significantly decreased. This phenomenon suggests that hydration reactions occurred during the soaking process, leading to changes in the shale structure and causing some particles to detach. Nonetheless, the length and diameter of the samples did not exhibit significant changes.

From the data in Table 2, we observe that after soaking the cores in distilled water, inorganic salt solutions of different concentrations, and inhibitor solutions, the acoustic transit time increased. This increase is due to the aqueous phase penetrating microcracks within the core, interacting with clay minerals, and inducing hydration, which alters the internal structure of the core. Such changes cause ultrasonic waves to take longer to traverse the core compared to the original sample, resulting in a reduced acoustic velocity given that the core length remains largely unchanged.

By horizontally comparing the data in the table, we find that as the concentration of the aqueous phase increases, the increment of the acoustic transit time (Δt_c_) shows a decreasing trend. Vertically comparing the data, it becomes apparent that the Δt_c_ values of cores soaked in inhibitor solutions are generally smaller than those soaked in inorganic salt solutions. Among the inorganic salts, the Δt_c_ values for KCl and CaCl_2_ are smaller than that for NaCl, indicating that KCl and CaCl_2_ solutions exhibit better inhibitory effects.

Within the inhibitor solutions, the Δt_c_ value after adding TIL-NH_2_ is smaller than those after adding polyamine DEM and polyetheramine. This suggests that the ability to inhibit shale hydration follows the order: TIL-NH_2_ > polyetheramine > DEM. This can be attributed to the strong adsorption of the imidazole cation structure and the primary amine functional group at the end of the side chain in TIL-NH_2_ onto clay particles. Such adsorption hinders the interaction between water molecules and clay particles, thereby achieving an efficient inhibitory effect on shale hydration. This experimental observation is consistent with the inhibitory performance evaluation results discussed earlier.

#### 2.3.6. Effect of Inhibitors on Shale Permeability Recoverability

**Figure 12:**
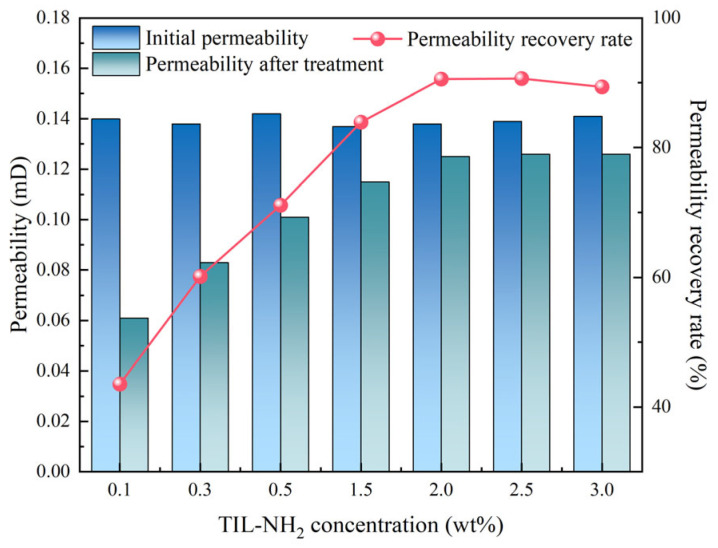
Variation in shale permeability recovery rates at different TIL-NH_2_ concentrations.

In this experiment, shale permeability recovery tests were conducted using TIL-NH_2_ solutions at various concentrations (0.1 wt%, 0.3 wt%, 0.5 wt%, 1.0 wt%, 1.5 wt%, 2.0 wt%, 2.5 wt%, and 3.0 wt%). The experimental results are shown in Figure 12.

According to the data presented in Figure 12, the permeability recovery rate generally increases with the concentration of TIL-NH_2_. However, after exceeding a concentration of 2.0 wt%, the recovery rate tends to stabilize and even shows a slight decline at 3.0 wt%. At lower concentrations (such as 0.1 wt% and 0.3 wt%), the inhibitory effect is limited, with recovery rates of only 43.57% and 60.14%, respectively. This indicates that the adsorption layer has not fully formed at these concentrations.

When the concentration reaches 0.5 wt%, the recovery rate significantly increases to 71.13%. Further increasing the concentration to 2.0 wt% leads to a recovery rate of 90.58%, suggesting that at this concentration, the adsorption layer and barrier stability have reached an optimal balance. However, when the concentration continues to increase to 2.5 wt% and 3.0 wt%, the recovery rates are 90.65% and 89.36%, respectively. The slight decrease at 3.0 wt% may be due to an aggregation effect of the adsorption layer caused by excessive concentration, which hinders further flow through the shale pores.

Analyzing the chemical structure of TIL-NH_2_, it can be inferred that TIL-NH_2_ effectively combines with the negative charges on the shale surface through electrostatic adsorption involving its imidazole groups and diamine groups. This interaction inhibits the swelling and migration of clay minerals. The long-chain polymer structure of TIL-NH_2_ forms a stable hydrophobic barrier on the shale surface, reducing the ingress of water molecules through steric hindrance effects. Additionally, hydrogen bonding between amino groups and hydroxyl or oxide groups on the shale further enhances adsorption stability, prolonging the inhibitor’s effectiveness within the shale pores.

These physicochemical mechanisms explain the observed increase in permeability recovery rate with increasing TIL-NH_2_ concentration. They also illustrate that at excessively high concentrations, the aggregation of the adsorption layer may affect pore fluidity, leading to a slight decrease in recovery rate.

#### 2.3.7. Analysis of the Dynamic Adsorption Behavior of Inhibitors

**Figure 13:**
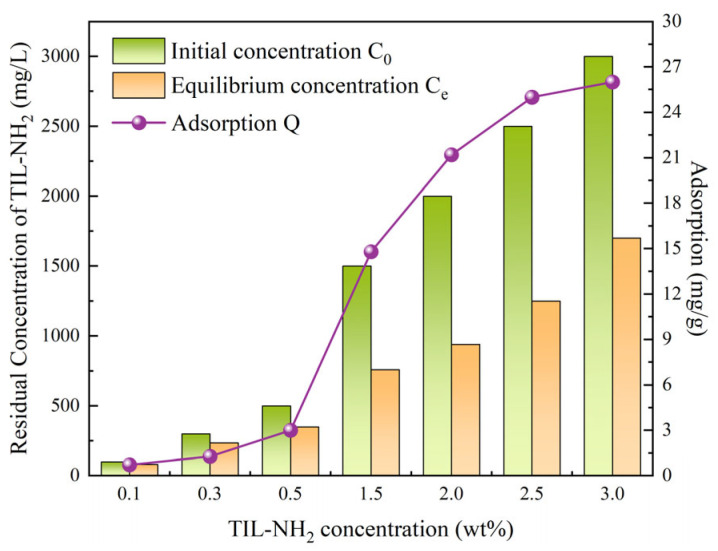
Dynamic adsorption as a function of TIL-NH_2_ concentration.

The dynamic adsorption behavior was measured using a fixed-bed experimental apparatus with shale core samples under simulated formation conditions. The experimental results are shown in Figure 13. (Experimental data points are represented by circles. The solid lines are simple interpolation lines used to illustrate general trends and do not represent any mathematical models or fitting functions).

From Figure 13, it can be observed that the adsorption amount of TIL-NH_2_ on the shale surface increases rapidly with rising concentration but slows down after exceeding 2.5 wt% and levels off at 3.0 wt%. At low concentrations (0.1 wt% to 0.5 wt%), the adsorption amounts are relatively low (ranging from 0.72 mg/g to 3.00 mg/g), indicating that adsorption at this stage is primarily surface physical adsorption and that the adsorption sites are not yet saturated. As the concentration increases to 2.0 wt%, the adsorption amount significantly rises to 21.20 mg/g. This suggests that TIL-NH_2_ molecules are fully interacting with the shale surface through electrostatic forces and hydrogen bonding, forming a relatively stable adsorption layer.

At concentrations between 2.5 wt% and 3.0 wt%, the adsorption amount approaches saturation (around 26.00 mg/g), indicating that the adsorption sites on the shale surface are nearly fully occupied. Excessive inhibitor molecules at higher concentrations may not further adsorb; instead, intermolecular repulsion might lead to a decrease in adsorption efficiency. Considering the chemical properties of TIL-NH_2_, its cationic imidazole groups and amino groups combine with the negatively charged clay minerals in shale through electrostatic interactions, enhancing adsorption stability. Additionally, the long-chain structure and multiple functional groups further promote the formation of chemical adsorption. The formation of the adsorption layer not only reduces the penetration of water molecules but also enhances the stability of the shale to a certain extent.

In summary, the dynamic adsorption behavior of TIL-NH_2_ exhibits clear concentration dependence and adsorption saturation characteristics, with the optimal adsorption effect occurring between 2.0 wt% and 2.5 wt%. This result confirms the excellent performance of TIL-NH_2_ in enhancing shale stability and reducing swelling, providing important theoretical support for its field application.

#### 2.3.8. Evaluation of Salt Resistance

**Figure 14:**
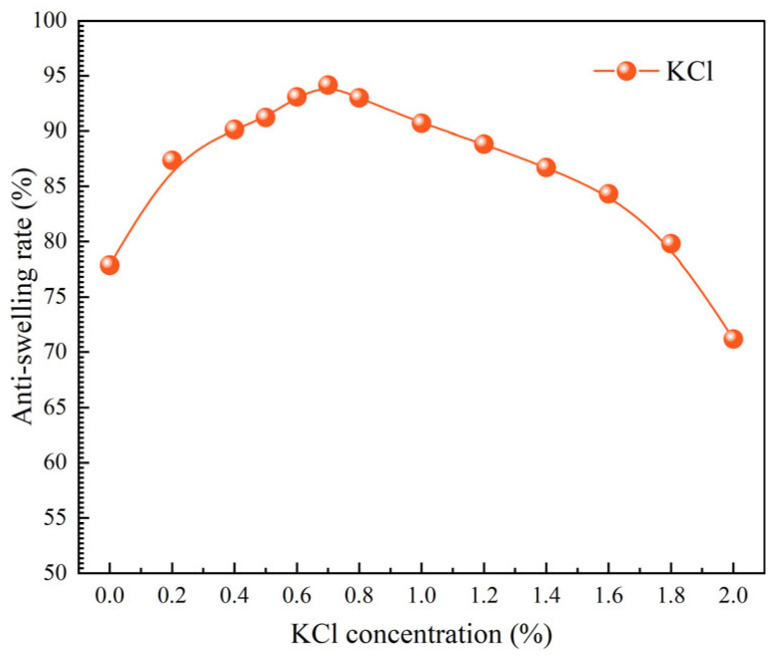
Influence of KCl concentration on the anti-swelling effectiveness of TIL-NH_2_.

In this section, a 1.2% TIL-NH_2_ solution was combined with varying concentrations of KCl to prepare blended solutions. The anti-swelling rates of these blended solutions were measured using the aforementioned method, and the results are presented in Figure 14. (Experimental data points are represented by circles. The solid lines are simple interpolation lines used to illustrate general trends and do not represent any mathematical models or fitting functions). As shown in the figure, the addition of a small amount of KCl to the TIL-NH_2_ solution significantly improved the anti-swelling rate. When the KCl concentration was less than 0.5%, the anti-swelling rate exhibited an upward trend, greatly surpassing that of the TIL-NH_2_ solution alone. However, when the KCl concentration exceeded 0.7%, the anti-swelling rate began to decline.

This phenomenon can be explained by the dual role of KCl in the system. On one hand, the addition of KCl helps neutralize the negative charges on illite, enhancing the adsorption of the blended system onto clay particles. On the other hand, due to the electrolyte effect, KCl can shield the ionic groups on the polymer chain, reducing the repulsive forces between like-charged ionic groups. This causes the flexible polymer chains to coil and aggregate, weakening the ability of the polymer hydration inhibitor to control the interlayer spacing of clay minerals. The polyelectrolyte effect intensifies with increasing KCl concentration within a certain range, thereby affecting the inhibitory performance of the blended system. These two factors are actually interrelated.

Table 3 presents the anti-swelling rates of various inhibitors at a mass fraction of 2%. Under identical experimental conditions, the data indicate that KCl exhibits the best anti-swelling effect. The anti-swelling rate of TIL-NH_2_ is similar to that of the commercial inhibitor Bop-3, demonstrating good anti-swelling performance. Although the commercial small-molecule quaternary ammonium salt inhibitor 132^#^ shows excellent anti-swelling effects, it has high toxicity, causes significant formation damage, and poses environmental pollution concerns.

#### 2.3.9. Environmental Friendliness Analysis

##### Biodegradability Performance

**Figure 15:**
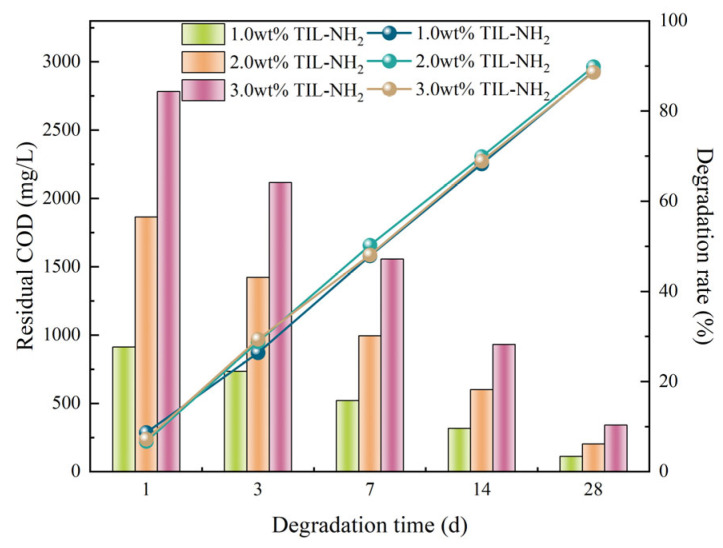
Biodegradation rates of different concentrations of TIL-NH_2_ as a function of time.

To evaluate the biodegradability of TIL-NH_2_, we employed the standard *BOD*_5_/*COD* testing method, combined with microbial degradation experiments under simulated environmental conditions, to determine its degradation rate over different time periods. The experimental results are shown in Figure 15.

The data in Figure 15 indicate that the degradation rates of all concentration groups increase significantly over time. In the early stage (1–3 days), the degradation rates are low (6.75–29.40%), suggesting limited microbial degradation efficiency as microorganisms adapt to the structure of TIL-NH_2_. In the middle stage (7–14 days), the degradation rates rise markedly, with the 2.0 wt% concentration group reaching 50.25–69.90%, indicating higher microbial metabolic activity at this concentration. In the later stage (28 days), the degradation rates approach 90%, demonstrating that TIL-NH_2_ can be almost completely degraded under experimental conditions.

Chemically, the imidazole and amino groups in the TIL-NH_2_ molecule provide easily degradable functional sites. These sites can be broken down by specific enzymes secreted by microorganisms into small-molecule intermediates, which are further metabolized into harmless substances like carbon dioxide and water. The degradation rates of high-concentration groups eventually converge with those of low-concentration groups, indicating that their degradation products do not inhibit microbial metabolism, further confirming the environmental friendliness of TIL-NH_2_. In summary, TIL-NH_2_ exhibits excellent biodegradability under the experimental conditions, providing solid scientific evidence and technical support for its practical application as an environmentally friendly shale inhibitor.

##### Toxicity Testing

To assess the toxicity of TIL-NH_2_, acute toxicity tests and microbial activity inhibition experiments were conducted, comparing it with the traditional shale inhibitor KCl solution. The experimental results are presented in Table 4 and Table 5.

Data from Table 4 show that at low concentrations (0.1–0.5 wt%), the acute toxicity of TIL-NH_2_ to zebrafish is significantly lower than that of KCl solution, with mortality rates of 0.0%, 5.8%, and 18.6%, respectively. At a concentration of 1.0 wt%, the mortality rate of TIL-NH_2_ is 44.7%, markedly lower than KCl’s 70.2%. The LC_50_ value of TIL-NH_2_ reaches 1080.3 mg/L, much higher than KCl’s 385.4 mg/L, indicating its extremely low acute toxicity and greatly enhanced safety.

Data in Table 5 show that at a concentration of 0.1 wt%, the inhibition rate of TIL-NH_2_ on microbial activity is only 0.30%, much lower than the 4.14% observed with KCl solution, indicating almost no effect on microorganisms. At higher concentrations of 1.0 wt% and 2.0 wt%, the inhibition rates of TIL-NH_2_ are 9.03% and 20.01%, respectively, significantly better than the 28.00% and 49.46% seen with KCl solution. This demonstrates that TIL-NH_2_ remains friendly to activated sludge even at high concentrations, showcasing significant environmental advantages.

By comparative analysis with the traditional shale inhibitor KCl solution, it can be concluded that TIL-NH_2_ is superior in both acute toxicity and microbial activity inhibition: its LC_50_ value is nearly three times that of KCl solution, exhibiting lower mortality rates in zebrafish, and having a significantly smaller impact on microbial activity.

Beyond its demonstrated biodegradability, the environmental impact of TIL-NH_2_ should be considered from a more holistic perspective. Future investigations could focus on the entire lifecycle of the inhibitor—from sourcing raw materials and synthesizing the polymer to managing its degradation products in the field environment. Utilizing renewable feedstocks, optimizing synthesis routes, and performing a full lifecycle assessment (LCA) could provide quantitative insights into its carbon footprint, energy consumption, and water use, ultimately guiding greener production practices. Moreover, the scalability of TIL-NH_2_’s synthesis to industrial levels is crucial for its widespread adoption. Preliminary indications suggest that large-scale production using commercially available monomers and conventional polymerization techniques is feasible, potentially reducing costs and resource inputs as economies of scale are realized. This approach can be further refined by exploring more sustainable manufacturing methods and improved purification processes, thereby minimizing environmental burdens while maintaining inhibitor efficacy.

When compared to traditional inhibitors such as KCl and NW-1, TIL-NH_2_ demonstrates markedly improved performance in several key metrics. At 1.2 wt%, TIL-NH_2_ reduces linear swelling by 65.69%, surpassing the swelling inhibition capabilities of commonly used salts. Under high-temperature conditions (140 °C), its shale cuttings rolling recovery rate reaches 88.12%, ensuring better stability than many existing polymeric and ionic liquid inhibitors. In terms of permeability recovery (90.58% at 2.0 wt%), TIL-NH_2_ effectively mitigates formation damage, further confirming its robust inhibitory functions.

What truly distinguishes this study from previous work is the dual-functional molecular design and the pronounced environmental benefits of TIL-NH_2_. In contrast to inhibitors relying on a single functional group, the imidazole cations and amino groups in TIL-NH_2_ operate synergistically, enhancing adsorption and inhibition under high-temperature, high-salinity conditions commonly encountered in deep and ultra-deep wells. Moreover, TIL-NH_2_’s rapid biodegradation (exceeding 90% within 28 days) and significantly reduced acute toxicity (with an LC_50_ value of 1080.3 mg/L compared to 385.4 mg/L for KCl) establish it as both a high-performance and environmentally responsible choice. Thus, TIL-NH_2_ not only meets engineering demands for improved wellbore stability but also aligns with the industry’s increasing emphasis on sustainability and ecological stewardship.

## 3. Discussion

After demonstrating TIL-NH_2_’s improved inhibitory performance, biodegradability, and lower toxicity compared to conventional inhibitors, this study underscores the inhibitor’s strong potential for advancing shale gas drilling fluid systems. Furthermore, by considering lifecycle factors and scalability, a pathway toward sustainable large-scale production and application emerges, aligning with increasingly rigorous environmental standards in oilfield operations.

Beyond these immediate applications in shale gas development, TIL-NH_2_’s multifunctional and environmentally favorable properties may hold value in other engineering domains. For instance, its effectiveness in controlling clay swelling and stabilizing fine particles suggests potential use in geotechnical engineering, where mitigating moisture-induced volume changes in clay-rich soils is critical for slope stability, foundation support, and embankment construction. Similarly, in the mining industry, incorporating TIL-NH_2_ into water management strategies could help maintain stable working conditions by reducing water infiltration and minimizing particle dispersion in clayey overburden. Additionally, infrastructure and soil remediation efforts could benefit from TIL-NH2’s environmentally friendly profile, as its incorporation might prevent soil erosion, enhance the longevity of roads and tunnels, and improve the stability of underground utilities installed in clay-dominated terrains. From an economic standpoint, the feasibility of large-scale production and application of TIL-NH_2_ is also promising. Although the introduction of bifunctional and ionic liquid moieties may initially suggest higher costs, the required raw materials and initiators are commercially available and can be sourced competitively. As production scales up, economies of scale are expected to narrow the cost gap relative to traditional inhibitors. Moreover, by improving wellbore stability, reducing non-productive time, and potentially extending drilling equipment life, TIL-NH_2_ may generate operational savings that offset any incremental material expenses. Additionally, its reduced environmental footprint may help operators meet stringent regulations and avoid potential penalties or remediation costs. Taken together, these factors contribute to a favorable cost-efficiency profile for TIL-NH_2_, reinforcing its value as a next-generation inhibitor suitable for contemporary drilling challenges.

In addition to demonstrating TIL-NH_2_’s superior inhibitory performance and environmental profile, the findings of this study provide a foundational framework for the next generation of shale inhibitors. For instance, our results indicate that incorporating bifunctional groups (e.g., imidazole cations and amino groups) can enhance adsorption and hydration suppression, while focusing on biodegradable polymeric structures can reduce ecological impacts. By applying these principles, future research can design innovative inhibitors tailored to specific geological conditions, improve compatibility with various drilling fluid systems, and ultimately contribute to more sustainable and efficient resource extraction.

## 4. Materials and Methods

### 4.1. Experimental Materials and Instruments

#### 4.1.1. Experimental Materials

1-Vinylimidazole, AR, Chengdu Kelong Chemical Reagent Factory (Chengdu, China); Acetonitrile, AR, Chengdu Kelong Chemical Reagent Factory; N-(2-Bromoethyl)-1,3-propanediamine dihydrobromide, AR, Shanghai Yuan Ye Bio-Technology Co., Ltd. (Shanghai, China); Anhydrous Ethanol, AR, Chengdu Kelong Chemical Reagent Factory; Azobisisobutyramidine Hydrochloride (V-50), AR, Shanghai Yuan Ye Bio-Technology Co., Ltd.; Triethylamine, AR, Chengdu Kelong Chemical Reagent Factory; KCl, AR, Chengdu Kelong Chemical Reagent Factory; Sodium Chloride NaCl, AR, Chengdu Kelong Chemical Reagent Factory; Polyetheramine, AR, Shanghai Yuan Ye Bio-Technology Co., Ltd.; Polyamine DEM, AR, Shanghai Yuan Ye Bio-Technology Co., Ltd.

#### 4.1.2. Experimental Apparatus

HH-6 type constant temperature water bath, Shanghai Yiheng Scientific Instrument Co., Ltd. (Shanghai, China); MK-6ST type digital six-speed rotational viscometer, Shandong Meike Instrument Co., Ltd. (Shanghai, China); 1705 type linear expansion meter, Qingdao Chuangmeng Instrument Co., Ltd. (Qingdao, China); XGRL-4 type high-temperature inverter roller heating furnace, Qingdao Haitongda Specialized Instrument Co., Analytical Instrument Company (Qingdao, China); ultrasonic testing system for rock core, self-researched.

### 4.2. Experimental Methods

**Figure 16:**
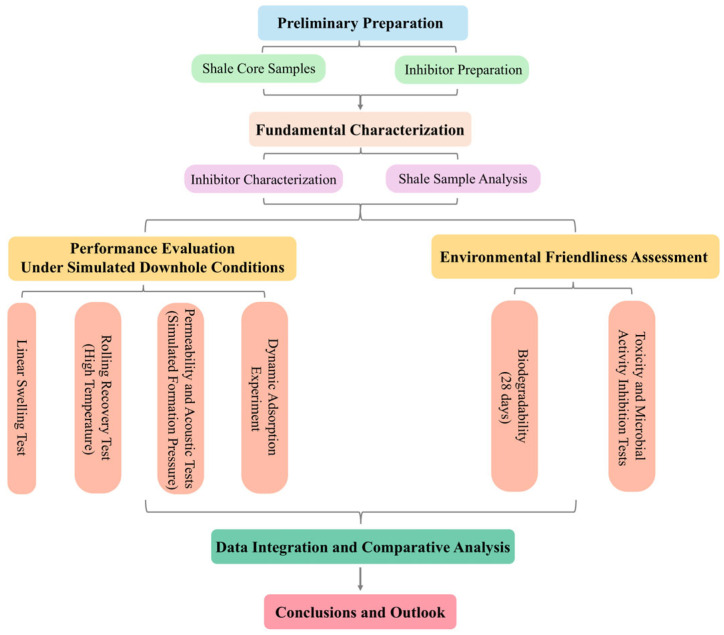
Flowchart summarizing the experimental design and workflow of this study.

Before outlining the specific experimental procedures, a flowchart illustrating the overall research design and methodology is presented in Figure 16. This schematic provides an integrated view of the steps undertaken, from sample preparation to performance evaluation and environmental assessment, ensuring a clear understanding of the logical sequence of experiments.

#### 4.2.1. Infrared Spectral Analysis

Firstly, a 4% shale powder base slurry was hydrated in a mixer for 24 h. Different mass ratios of TIL-NH_2_ were then added and stirred for an additional 24 h to prepare inhibitor solutions of varying concentrations. Subsequently, 45 mL of the solution was placed in an 8000 rpm centrifuge for 5 min. The precipitate was gently washed three times with a total of 60 mL of deionized water, then dried in an oven at 105 °C for 4 h, and finally ground into a dry powder. A mixture of 2 mg of this powder and 200 mg of KBr particles was uniformly blended and pressed into pellets under a pressure of 17 MPa for infrared spectroscopy analysis.

The infrared spectra were recorded using a Nicolet 6700 Fourier-transform infrared (FTIR) spectrometer. Samples were scanned over a wavenumber range of 500 cm^−1^ to 4000 cm^−1^ with a step frequency of 1.928 cm^−1^. The wavenumber accuracy was better than 0.005 cm^−1^, and linearity deviation was less than 0.07%.

#### 4.2.2. Nuclear Magnetic Resonance Spectral Test

Approximately 12 mg of TIL-NH_2_ was dissolved in 0.5 mL of dimethyl sulfoxide-d_6_ (DMSO-d_6_), using tetramethylsilane (TMS) as the internal standard. The chemical shifts were measured using an NMR spectrometer, with frequency resolution ≤ 0.005 Hz and phase resolution ≤ 0.01 during the measurement.

#### 4.2.3. Rock Chip Rolling Recovery Experiments

To effectively control the hydration effect of drilling fluids on shale cuttings and consider the influence of geological conditions on shale hydration, the thermal rolling recovery rate was used to evaluate the inhibitory performance of TIL-NH_2_ under simulated high-temperature conditions in the laboratory. The thermal rolling recovery rate refers to the ratio of the mass of cuttings retained on the sieve after high-temperature and high-pressure aging to the initial mass of the cuttings. It reflects the inhibitor’s ability to prevent the hydration dispersion of cuttings—the higher the recovery rate, the better the inhibition effect.

The thermal rolling recovery rate of Longmaxi Formation shale cuttings in different concentrations of TIL-NH_2_ solution was measured as follows: ① Sample Preparation: Place 50 g of dried shale cuttings (6–10 mesh) into a sealed container and add 350 mL of distilled water or TIL-NH_2_ solution. ② Aging Process: Seal the container and place it in a BRGL-7 variable-frequency roller heating oven. The temperature was set within a range of 120–200 °C, with a specific focus on 140 °C for detailed analysis. This temperature range is designed to simulate bottom-hole temperatures encountered in shale gas reservoirs, particularly deep formations such as the Longmaxi Formation, where temperatures can reach or exceed 140 °C. The temperature of 140 °C was specifically chosen as it represents a common bottom-hole temperature in deep shale gas reservoirs and is frequently used in comparable studies for benchmarking purposes. Higher temperatures within the testing range were utilized to evaluate the thermal stability of the inhibitor. The samples were aged for 16 h, consistent with industry standards and allowing ample time to observe the hydration and dispersion of the cuttings at elevated temperatures. ③ Screening and Washing: After aging, pour the mixture over a 40-mesh sieve and rinse the retained cuttings with tap water to remove any adhering fines. ④ Drying and Weighing: Dry the retained cuttings at 105 °C for 24 h to obtain the mass *M*_r_. ⑤ Calculation: The thermal rolling recovery rate is calculated using Equation (1):

(1) $$Recovery\;Rate=\frac{M_{r}}{50g}\times 100\%$$

#### 4.2.4. Analysis of Anti-Expansion Properties

According to the standard SY/T 5971-94 [41] “Evaluation Methods for Clay Stabilizers Used in Water Injection”, the anti-swelling rate was measured. Specifically, 2 g of shale powder dried to constant weight at 100 °C was placed into a graduated test tube, and 10 mL of the test solution was added. After thorough stirring, the mixture was allowed to stand for 2 h, followed by centrifugation at 2000 rpm for 15 min to separate the solid and liquid phases. The height of the swollen shale powder was then measured. The anti-swelling rate *HR* was calculated using Equation (2):

(2) $$HR=\frac{H_{2}-H_{1}}{H_{2}-H_{0}}$$

where $H_{2}$ is the height of shale powder expansion in water, mm;

$H_{1}$ is the height of shale powder swelling in polymer solution, mm;

$H_{0}$ is the height of shale powder expansion in kerosene, mm.

#### 4.2.5. Core Acoustic Time Difference Analysis

Using Longmaxi Formation shale from the Weiyuan area as an example, the effects of soaking standard shale samples in different types and concentrations of inorganic salts, inhibitors, and distilled water were experimentally investigated. The soaking duration was 72 h under ambient temperature and pressure conditions. During the experiment, a 100 kHz longitudinal ultrasonic transducer was used for acoustic testing, with the confining pressure set at 0.3 MPa. This lower confining pressure was selected to facilitate the observation of microstructural changes within the shale samples resulting from hydration. While this confining pressure does not fully replicate in-situ stress conditions, this methodology is widely accepted for comparatively assessing the impact of different fluids on rock integrity. Acoustic measurements were conducted before and after soaking to compare changes in the samples’ properties.

#### 4.2.6. Penetration Rate Resilience Analysis

Permeability tests were conducted using a permeability testing apparatus, simulating a formation pressure of 20 MPa and a temperature of 80 °C. The pressure of 20 MPa is representative of reservoir pressures found in many shale gas formations, including the Longmaxi Formation. The temperature of 80 °C was selected considering that subsurface temperatures are typically elevated compared to surface temperatures, providing a reasonable simulation of downhole thermal conditions. The procedure was as follows: ① Sample Preparation: Natural shale cores with a diameter of 2.5 cm and a height of 5 cm were selected. After cleaning and drying, the cores were saturated with simulated formation water. ② Initial Permeability Test: The samples were placed in the testing apparatus, and initial permeability was measured using simulated formation water. ③ Treatment with TIL-NH_2_: Different concentrations of TIL-NH_2_ solution were injected, and the samples were treated for 24 h. ④ Permeability Recovery Measurement: After treatment, permeability was measured again using simulated formation water. The permeability recovery rate was calculated using the following: (permeability recovery rate = post-treatment permeability/initial permeability × 100%).

#### 4.2.7. Adsorption Behavior Analysis

The experimental steps were as follows: ① Sample Preparation: Natural shale cores were prepared into cylindrical samples with a diameter of 2.5 cm and a height of 5 cm. After cleaning and drying, they were placed in a fixed-bed reactor. ② Experimental Parameters: TIL-NH_2_ solutions of different concentrations (0.1 wt%, 0.3 wt%, 0.5 wt%, 1.5 wt%, 2.0 wt%, 2.5 wt%, 3.0 wt%) were injected. The flow rate was controlled at 1.0 mL/min, the temperature was set at 80 °C, and the pressure was 20 MPa. The dynamic adsorption time was set to 8 h. ③ Adsorption Capacity Calculation: By recording the concentration changes of the effluent, the adsorption capacity of TIL-NH_2_ per unit mass of shale (mg/g) was calculated using the formula:

(3) $$Q=\frac{\left(C_{0}-C_{e}\right)V}{W}$$

where Q is the adsorption amount (mg/g); $C_{0}$ is the inlet concentration (mg/L); $C_{e}$ is the equilibrium concentration (mg/L); V is the inlet volume (L); W is the core mass (g).

#### 4.2.8. Environmental Friendliness Test

##### Biodegradability Test

① Sample Preparation: TIL-NH_2_ was dissolved in deionized water to prepare solutions with mass concentrations of 1.0 wt%, 2.0 wt%, and 3.0 wt%. Three parallel samples were set for each group. ② Experimental Conditions: Samples were placed in a simulated environmental reactor under conditions of 30 °C and pH 7.0. Air was supplied, and a mixed microbial consortium was added (initial bacterial concentration of 10^6^ CFU/mL). ③ Degradation Rate Measurement: Samples were taken on days 1, 3, 7, 14, and 28 to measure biochemical oxygen demand over 5 days (*BOD*_5_) and chemical oxygen demand (*COD*). The initial *COD* values for the 1.0 wt%, 2.0 wt%, and 3.0 wt% TIL-NH_2_ solutions were 1000 mg/L, 2000 mg/L, and 3000 mg/L, respectively. The degradation rate was calculated using the following equation:

(4) $$D=\frac{\left(COD_{i}-COD_{t}\right)}{COD_{i}}\times 100\%$$

where D is the degradation rate (%); $COD_{i}$ is the chemical oxygen demand (*COD*) of the sample measured at the beginning of the degradation experiment (mg/L); $COD_{t}$ is the chemical oxygen demand (*COD*) of the sample measured at time t of the degradation experiment.

##### Acute Toxicity Test

① Test Organism: Zebrafish (*Danio rerio*) were selected as the test organisms and exposed to different concentrations of TIL-NH_2_ solutions (0.1 wt%, 0.3 wt%, 0.5 wt%, 1.0 wt%, 2.0 wt%) for a duration of 96 h. ② Measurement Indicators: The mortality rate of zebrafish was recorded over the 96-h period to calculate the median lethal concentration (LC_50_, mg/L).

##### Microbial Activity Inhibition Test

① Test Organism: A mixed microbial consortium from activated sludge was used. TIL-NH_2_ solutions with concentrations of 1.0 wt% and 2.0 wt% were prepared and mixed with the sludge. The temperature was set at 30 °C, and pH was maintained at 7.0. ② Measurement Indicators: The metabolic activity of the microbial consortium was assessed by measuring *BOD*_5_. The inhibition rate of TIL-NH_2_ on microbial activity was calculated using the following:

(5) $$IR=\frac{{{BOD}_{5}}_{i}-{{BOD}_{5}}_{dw}}{{{BOD}_{5}}_{i}}\times 100\%$$

where IR-inhibition rate (%); ${{BOD}_{5}}_{i}$-value of initial *BOD*_5_ (mg/L); ${{BOD}_{5}}_{dw}$-value of treated *BOD*_5_ (mg/L).

## 5. Conclusions

Excellent Inhibitory Performance with High Temperature and Salt Resistance: The novel inhibitor TIL-NH_2_ exhibits significant shale hydration inhibition capabilities due to its bifunctional group structure. Experimental results demonstrate that at a concentration of 1.2 wt%, TIL-NH_2_ reduces the linear swelling height of shale by 65.69%. Under conditions of 140 °C, its shale cuttings rolling recovery rate reaches 88.12%, showcasing excellent high-temperature and salt-resistant properties that meet the engineering demands of complex downhole environments.

Remarkable Dynamic Adsorption and Permeability Recovery Performance: The dynamic adsorption behavior of TIL-NH_2_ shows concentration dependency, reaching saturation at 2.5 wt% with an adsorption amount of 26.00 mg/g. This forms a stable adsorption layer that significantly inhibits shale hydration. Additionally, in permeability recovery experiments, TIL-NH_2_ at a concentration of 2.0 wt% achieves a shale permeability recovery rate of 90.58%, indicating a notable effect in repairing damage to shale formations.

Excellent Environmental Friendliness: TIL-NH_2_ demonstrates good environmental friendliness, with a degradation rate exceeding 90% within 28 days, indicating rapid biodegradability. Moreover, its acute toxicity is significantly lower than that of the traditional inhibitor KCl, with an LC50 value of 1080.3 mg/L. This suggests that TIL-NH_2_ not only meets the engineering requirements for high-efficiency inhibition but also reduces environmental burdens, providing reliable support for the promotion of green drilling fluid systems.

## Figures and Tables

**Table 1:** Base parameters of Longmaxi shale rock samples before and after soaking.

Experimental Group	Immersion Solution	Protolith			After Immersion			
		Drying			Undried			After Drying
		Lengths	Calibre	Mass (In Physics)	Lengths	Calibre	Mass (In Physics)	Mass (In Physics)
		(mm)	(mm)	(g)	(mm)	(mm)	(g)	(g)
	distilled water	50.22	25.20	61.17	50.23	25.20	62.58	60.84
NaCl	0.3 mol/L	50.13	25.22	62.97	50.28	25.15	63.90	62.59
	0.6 mol/L	50.24	25.21	63.22	50.21	25.25	64.18	62.87
	1.2 mol/L	50.28	25.24	65.15	50.32	25.24	66.04	64.69
KCl	0.3 mol/L	50.30	25.13	64.96	50.21	25.08	65.56	64.50
	0.6 mol/L	50.24	25.21	62.98	50.20	25.25	64.01	62.60
	1.2 mol/L	50.20	25.22	63.07	50.14	25.28	64.17	62.76
CaCl_2_	0.3 mol/L	50.28	25.22	61.20	50.34	25.27	62.07	60.88
	0.6 mol/L	50.16	25.21	63.45	50.20	25.23	64.13	63.15
	1.2 mol/L	50.45	25.11	61.44	50.19	25.18	61.02	60.30
DEM	0.3 mol/L	50.08	25.21	62.21	50.26	25.20	62.58	61.34
	0.6 mol/L	50.17	25.18	62.87	50.05	25.25	63.95	62.59
	1.2 mol/L	50.33	25.23	63.32	50.12	25.23	64.33	62.87
polyetheramine	0.3 mol/L	50.02	25.21	65.25	50.12	25.56	66.27	64.70
	0.6 mol/L	50.32	25.27	64.17	50.20	25.45	65.61	64.51
	1.2 mol/L	50.35	25.28	62.26	50.14	25.39	64.21	62.55
TIL-NH_2_	0.3 mol/L	50.17	25.36	63.30	50.15	25.47	64.89	62.46
	0.6 mol/L	50.20	25.24	61.22	50.14	25.31	62.37	60.91
	1.2 mol/L	50.23	25.14	63.51	50.12	25.25	64.38	63.20

**Table 2:** Acoustic time difference of Longmaxi shale rock samples before and after soaking.

Experimental Group	Solution Concentration	△tc-100 khz Pre-Soaking	△tc-100 khz After Immersion	△tc-100 khz Difference (The Result of Subtraction)
	distilled water	237.33	255.79	18.46
NaCl	0.3 mol/L	281.83	300.82	18.99
	0.6 mol/L	277.52	294.90	17.38
	1.2 mol/L	279.13	287.15	8.02
KCl	0.3 mol/L	253.44	273.88	20.44
	0.6 mol/L	248.97	261.83	12.86
	1.2 mol/L	256.21	262.42	6.21
CaCl_2_	0.3 mol/L	288.31	303.98	15.67
	0.6 mol/L	272.28	282.67	10.39
	1.2 mol/L	278.33	284.51	6.18
DEM	0.3 mol/L	276.27	291.51	15.24
	0.6 mol/L	254.48	265.35	10.87
	1.2 mol/L	268.03	273.45	5.42
polyetheramine	0.3 mol/L	290.31	304.65	14.34
	0.6 mol/L	310.25	320.18	9.93
	1.2 mol/L	324.57	329.84	5.27
TIL-NH_2_	0.3 mol/L	287.38	299.93	12.55
	0.6 mol/L	281.27	289.32	8.05
	1.2 mol/L	300.16	304.42	4.26

**Table 3:** Anti-expansion rate of different inhibitors.

Suppressant	KCl	132	Bop-3	TIL-NH_2_
Inflatability (%)	94.95	82.90	80.00	80.35

**Table 4:** Comparison of acute toxicity test data between TIL-NH_2_ and KCl.

Type of Inhibitor	Concentration (wt%)	Zebrafish Mortality (%)	LC_50_ (mg/L)
TIL-NH_2_	0.1	0.0	-
	0.3	5.8	-
	0.5	18.6	-
	1.0	44.7	1080.3
	2.0	65.4	-
KCl solution	0.1	5.0	-
	0.3	20.3	-
	0.5	45.0	-
	1.0	70.2	385.4
	2.0	89.7	-

**Table 5:** Comparison of microbial activity inhibition experimental data between TIL-NH_2_ and KCl.

Type of Inhibitor	Concentration (wt%)	Initial *BOD*_5_ (mg/L)	*BOD* After Treatment_5_ (mg/L)	Inhibition Rate (%)
TIL-NH_2_	0.1	202.4	201.8	0.30
	1.0	201.7	183.5	9.03
	2.0	200.9	160.7	20.01
KCl solution	0.1	200.8	192.5	4.14
	1.0	198.3	142.8	28.00
	2.0	199.2	100.6	49.46

## Data Availability

The figures and tables used to support the findings of this study are included in the article.
